# The complete chloroplast genome sequence of *Mytilaria laosensis*

**DOI:** 10.1080/23802359.2019.1688118

**Published:** 2019-11-08

**Authors:** Yi Wang, Yunqing Li, Xiaolong Yuan, Jinfeng Zhang

**Affiliations:** Laboratory of Forest Plant Cultivation and Utilization, Yunnan Academy of Forestry, Kunming Yunnan, People's Republic of China

**Keywords:** *Mytilaria laosensis*, chloroplast, Illumina sequencing, phylogenetic analysis

## Abstract

The first complete chloroplast genome (cpDNA) sequence of *Mytilaria laosensis* was determined from Illumina HiSeq pair-end sequencing data in this study. The cpDNA is 159,941 bp in length, contains a large single-copy region (LSC) of 89,016 bp and a small single-copy region (SSC) of 18,127 bp, which were separated by a pair of inverted repeats (IR) regions of 26,399 bp. The genome contains 130 genes, including 85 protein-coding genes, 8 ribosomal RNA genes, and 37 transfer RNA genes. The overall GC content of the whole genome is 37.9%, and the corresponding values of the LSC, SSC, and IR regions are 35.9, 32.8, and 43.1%, respectively. Further phylogenomic analysis showed that *M. laosensis* and *Chunia bucklandioides* clustered in a clade in family Hamamelidaceae.

*Mytilaria laosensis* is the species of the genus *Mytilaria* within the family Hamamelidaceae. It is an important broad-leaved tree species indigenous to the tropical and subtropical areas of southeast Asia and south China (Yu et al. 2018). It is famous for its good adaptability, straight and full stem form, good wood quality, fast-growing (Tang et al. 2018). It has been widely used in the establishment of ecological benefit-oriented forests as well as fast-growing and high-yield plantations (Chen et al. 2012). It is also a high-quality raw material for building, furniture, papermaking and wood-based panels (Bai et al. 2011; Yu et al. 2019). However, there have been no genomic studies on *M. laosensis*.

Herein, we reported and characterized the complete *M. laosensis* plastid genome (MN106252). One *M. laosensis* individual (specimen number: 201807015) was collected from Puwen, Yunnan Province of China (23°76′12″ N, 101°27′15″ E). The specimen is stored at Yunnan Academy of Forestry Herbarium, Kunming, China, and the accession number is YAFH0012744. DNA was extracted from its fresh leaves using DNA Plantzol Reagent (Invitrogen, Carlsbad, CA, USA).

Paired-end reads were sequenced by using Illumina HiSeq system (Illumina, San Diego, CA). In total, about 22.6 million high-quality clean reads were generated with adaptors trimmed. Aligning, assembly, and annotation were conducted by CLC de novo assembler (CLC Bio, Aarhus, Denmark), BLAST, GeSeq (Tillich et al. 2017), and GENEIOUS v 11.0.5 (Biomatters Ltd, Auckland, New Zealand). To confirm the phylogenetic position of *M. laosensis*, other seven species of family *Hamamelidaceae* from NCBI were aligned using MAFFT v.7 (Katoh and Standley 2013) and maximum likelihood (ML) bootstrap analysis was conducted using RAxML (Stamatakis 2006); bootstrap probability values were calculated from 1000 replicates. *Liquidambar formosana* (KC588388) was served as the out-group.

The complete *M. laosensis* plastid genome is a circular DNA molecule with the length of 159,941 bp, contains a large single-copy region (LSC) of 89,016 bp and a small single-copy region (SSC) of 18,127 bp, which were separated by a pair of inverted repeat (IR) regions of 26,399 bp. The overall GC content of the whole genome is 37.9%, and the corresponding values of the LSC, SSC, and IR regions are 35.9, 32.8, and 43.1%, respectively. The plastid genome contained 130 genes, including 85 protein-coding genes, 8 ribosomal RNA genes, and 37 transfer RNA genes. Phylogenetic analysis showed that *M. laosensis* and *Chunia bucklandioides* clustered in a unique clade in family *Hamamelidaceae* (Figure 1). The determination of the complete plastid genome sequences provided new molecular data to illuminate the *Hamamelidaceae* evolution.

**Figure 1.**
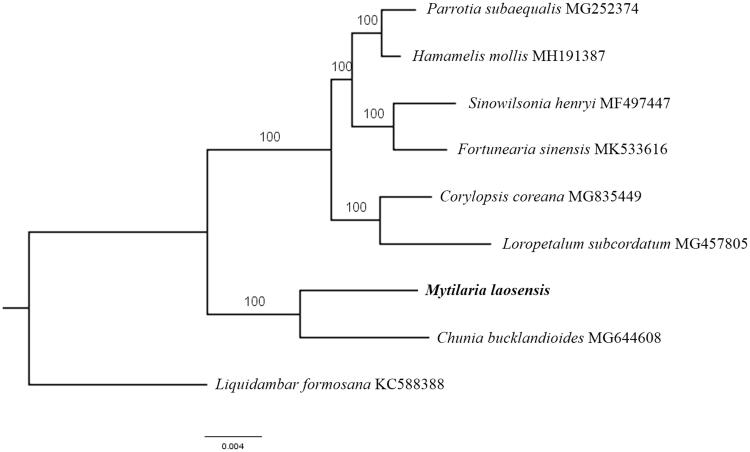
The maximum-likelihood tree based on the eight chloroplast genomes of *Hamamelidaceae*. The bootstrap value based on 1000 replicates is shown on each node.
